# Maintenance and reappearance of extremely divergent intra-host HIV-1 variants

**DOI:** 10.1093/ve/vey030

**Published:** 2018-12-04

**Authors:** Joel O Wertheim, Alexandra M Oster, Ben Murrell, Neeraja Saduvala, Walid Heneine, William M Switzer, Jeffrey A Johnson

**Affiliations:** 1 Department of Medicine, University of California, San Diego, USA; 2 Division of HIV/AIDS Prevention, Centers for Disease Control and Prevention, Atlanta, USA; 3 ICF International, Atlanta, USA

**Keywords:** HIV, superinfection, molecular evolution, genetic variation

## Abstract

Understanding genetic variation in human immunodeficiency virus (HIV) is clinically and immunologically important for patient treatment and vaccine development. We investigated the longitudinal intra-host genetic variation of HIV in over 3,000 individuals in the US National HIV Surveillance System with at least four reported HIV-1 polymerase (*pol*) sequences. In this population, we identified 149 putative instances of superinfection (i.e. an individual sequentially infected with genetically divergent, polyphyletic viruses). Unexpectedly, we discovered a group of 240 individuals with consecutively sampled viral strains that were >0.015 substitutions/site divergent, despite remaining monophyletic in the phylogeny. Viruses in some of these individuals had a maximum genetic divergence approaching that found between two random, unrelated HIV-1 subtype-B *pol* sequences within the US population. Individuals with these highly divergent viruses tended to be diagnosed nearly a decade earlier in the epidemic than people with superinfection or virus with less intra-host genetic variation, and they had distinct transmission risk factor profiles. To better understand this genetic variation in cases with extremely divergent, monophyletic viruses, we performed molecular clock phylogenetic analysis. Our findings suggest that, like Hepatitis C virus, extremely divergent HIV lineages can be maintained within an individual and reemerge over a period of years.

## 1. Introduction

Intra-host genetic variation found in human immunodeficiency virus (HIV) infection is produced by complex evolutionary dynamics, including rapid evolution and genetic recombination (Shankarappa et al. 1999; Zanini et al. 2015). Within the HIV-1 protease and polymerase (*pol*) genomic region commonly used for drug resistance testing, the maximum divergence between intra-host variants tends to be <0.01–0.02 substitutions/site (Hightower et al. 2013; Poon et al. 2015; Zanini et al. 2015). In North America, typical HIV-1 subtype B strains from different individuals are between 0.03 and 0.08 substitutions/site divergent (Poon et al. 2016; Wertheim et al. 2017a). Within a given individual, HIV diversity, especially in the envelope (*env*) region, tends to be periodically purged by selective sweeps (Shankarappa et al. 1999; Laird Smith et al. 2016; Landais et al. 2017).

HIV-1 superinfection occurs when an individual is sequentially infected with HIV from two different sources (Ramos et al. 2002; Smith et al. 2004; Smith, Richman, and Little 2005; Koning et al. 2013), which are often identified through a polyphyletic relationship in a phylogenetic tree (Wagner et al. 2014). Viral population subsequent to superinfection can reflect a mixture of the descendants of the two infecting strains, recombinant products of the infecting strains, or a single predominant strain. Superinfection can potentially affect the host immune response, disease progression, antiretroviral therapy (ART) and vaccine design and efficacy (Koelsch et al. 2003; Smith et al. 2005; Ronen et al. 2014; Wagner et al. 2017). There is a high incidence rate of superinfection: 4.96 per 100 person-years in high-risk cohorts of men who have sex with men (MSM) (Wagner et al. 2014) and 2.2 per 100 person-years in people who inject drugs (PWIDs) (Hu et al. 2005).

We investigated the longitudinal intra-host genetic variation of HIV *pol*, with the intent of characterizing cases of superinfection in a US National HIV Surveillance System (NHSS). We employed a combined phylogenetic and genetic distance-based approach. As part of this investigation, we discovered a group of individuals with extremely divergent viral genotypes that were monophyletic in an HIV phylogeny. This finding suggests that extremely divergent HIV *pol* lineages can be maintained over the course of prolonged infection. Here, we characterize this unexpected pattern of HIV genetic variation and discuss implications for the detection of HIV molecular transmission clusters in a surveillance context.

## 2. Methods

### 2.1 Epidemiologic and sequence data

HIV-1 *pol* sequences reported to the US NHSS from 2000 through Fall 2015 were included in the study (see Oster et al. 2015, for a description of the development of this sequence database). Sequence and epidemiological data were included in our analysis if they were from an individual with at least four longitudinally reported *pol* sequences, each sampled at least 30 days apart. Sequences reported to the NHSS are generated using bulk Sanger sequencing and this consensus sequence represent a snapshot of intra-host viral diversity at the time of sampling. All sequences were required to be a minimum of 500 nucleotides in length. In total, 3,655 people met these criteria, totaling 17,688 sequences.

### 2.2 Subtype classification and characterization of drug resistance associated mutations

HIV-1 subtypes and circulating recombinant forms were classified using a local installation of COMET v.1 (COntext-based Modeling for Expeditious Typing) (Struck et al. 2014). Non-B subtypes were included in phylogenetic analysis for rooting purposes, necessary to establish monophyly versus polyphyly. However, sequences from individuals with non-B subtypes (*n* = 152 individuals) were excluded from subsequent analyses given the variable substitution rates across HIV subtypes (Abecasis, Vandamme, and Lemey 2009; Wertheim, Fourment, and Kosakovsky Pond 2012). Drug resistance associated mutations (DRAMs) were identified using the HIV Drug Resistance Database via the Sierra Web Server 2.0 (https://hivdb.stanford.edu/page/webservice/) (Liu and Shafer 2006).

### 2.3 Calculating viral genetic divergence

To determine intra-host genetic distance, we used a local installation of HIV-TRACE (HIV TRAnsmission Cluster Engine) (Kosakovsky Pond et al. 2018). Briefly, HIV-1 *pol* sequences were aligned in pairwise fashion to a reference sequence (HXB2; coordinates 2,253–3,749). TN93 (Tamura and Nei 1993) genetic distances were calculated among each pair of sequences from a given individual. Unlike in previous HIV-TRACE analyses of the NHSS data, all distances between nucleotide ambiguities were resolved (e.g. Y is 0 substitutions from both C and T) to lessen the likelihood that sequences from mixed infections or those of poor quality would spuriously be flagged as being highly divergent. For each person, we determined if consecutively sampled genotypes were more than 0.015 nucleotide substitutions/site divergent. This distance threshold was selected based on previous analysis of local and national HIV surveillance data in the USA (Oster et al. 2015; Wertheim et al. 2016; Wertheim et al. 2017a). In an HIV-1 surveillance context, if two individuals have HIV genetic sequences that are ≤0.015 nucleotide substitutions/site divergent, this similarity implies a direct or indirect epidemiological linkage (Wertheim et al. 2017a). Therefore, we queried the database for instances in which consecutive sequences from within a single individual would not be suggestive of epidemiological linkage.

### 2.4 Phylogenetic analysis

A maximum-likelihood phylogenetic tree was inferred from the 17,688 sequences using FastTree2 under a GTR + CAT20 model (Price, Dehal, and Arkin 2010). Our inclusion criteria are biased towards individuals who are ART-experienced; therefore, we excluded 108 codons associated with DRAMs (Wheeler et al. 2010), as convergent evolution towards drug resistance can confound phylogenetic inference (Lemey et al. 2005). We used the ETE3 Toolkit (Huerta-Cepas, Serra, and Bork 2016) to determine whether the sequences from each of the 3,503 people with pure-subtype B virus were monophyletic or polyphyletic in the inferred phylogeny. A polyphyletic arrangement implies superinfection (Koelsch et al. 2003; Smith et al. 2004; Wagner et al. 2014), whereas monophyly suggests a single origin of infection (or potentially superinfection from a closely related source; see Section 4).

### 2.5 Regression analysis

Based on the genetic distance and phylogenetic analysis, we identified three populations for analysis: (1) monophyletic viruses with no consecutive strains exceeding 0.015 substitutions/site divergence [*n* = 2, 914 individuals], (2) monophyletic viruses with at least one consecutive strain exceeding 0.015 substitutions/site divergence [*n* = 240 individuals], and (3) polyphyletic viruses with at least one consecutive strains exceeding 0.015 substitutions/site divergence [*n* = 149 individuals]. We excluded individuals with monophyletic virus in which a single virus was >0.015 substitutions/site from all other viruses in that person, because these instances cannot be easily distinguished from poor sequence quality (*n* = 136 individuals). We also excluded individuals with non-monophyletic virus where the maximum genetic distance was <0.015 substitutions/site, because these instances cannot be easily distinguished from transmission within a local transmission cluster or poor resolution in a large phylogenetic tree (*n* = 64 individuals). Our final dataset comprised 3,303 individuals.

We performed multivariate multinomial logistic regression analysis to investigate differences in these three populations. This regression analysis included year of diagnosis; transmission risk factor (MSM, PWIDs, people reporting high-risk heterosexual contact [heterosexual], perinatal, and other risk factors); and presence of common DRAMs (limited to M184V, K65R, K103N, Y181C, G190A, and L90M). MSM who reported injection drug use were classified as PWID. Regarding DRAMs, we considered mixed populations (i.e. sequence ambiguities indicating the presence of both DRAM and wild-type variants) to be presence of a DRAM.

### 2.6 Molecular clock analysis

We explored the viral dynamics in individuals with monophyletic, extremely divergent intra-host viruses. Sixty-three individuals had a maximum intra-host distance of ≥0.025 substitutions/site; we performed Bayesian molecular clock phylogenetic analysis on the eleven of these individuals with ≥10 viral genotypes using BEAST 1.8.2 (Drummond et al., 2012). For each individual, two independent runs of 5 million generations were performed, sampling every 500 generations and removing the first 10% as burn-in. A TN93 substitution model was implemented, including gamma rate variation. Month and year of genotype sampling was used to calibrate the molecular clock. Given the limited signal for calibrating a molecular clock in HIV trees of this size, we imposed a highly informative prior distribution on the substitution rate parameter of the strict molecular clock model, with a mean of 1.22 × 10^−3^ substitutions/site/year and standard deviation of 1 × 10^−^^6^. This calibration comes from previous molecular dating using NHSS data (Wertheim et al. 2017b). A Bayesian Skyline coalescent prior with two steps was used. Convergence was assessed using TRACER 1.7 (Rambaut et al. 2018). We also performed maximum likelihood phylogenetic inference on these eleven trees using RAxML (Stamatakis 2014). The BEAST and RAxML phylogenies are available as Supplementary Material.

### 2.7 Recombination detection

Using the recombination detection program (RDP) in RDP4 we scanned for genetic recombination in 134 sequences from the 11 individuals with monophyletic viruses with the greatest intra-host genetic divergence (Martin et al. 2010).

## 3. Results

### 3.1 Scan for superinfection

We interrogated the NHSS for evidence of superinfection. We identified instances in which virus from within a single individual was polyphyletic in the phylogeny and had a consecutively sampled virus that was >0.015 substitutions/site divergent. Of the 3,303 individuals infected with pure subtype B strains, 149 (4.5%) met these criteria for defining superinfection. Of these 149 individuals, only 9 individuals had viruses in which the divergent virus was genetically similar (≤0.015 substitutions/site) to another virus in the same host.

### 3.2 Within-host genetic divergence

To investigate patterns of longitudinal viral divergence, we identified a group for whom there was no evidence of superinfection: individuals with monophyletic virus in which consecutive viruses are never more than 0.015 substitutions/site divergent from the previous virus. Of the 3, 303 individuals infected with pure subtype B strains, we found 2,914 individuals (88.2%) who met these criteria. Unexpectedly, we found 240 individuals (7.3%) with monophyletic virus in which one or more consecutively sampled viruses was >0.015 substitutions/site from the previously sampled virus.

### 3.3 Maximum within-host genetic distance

**Figure 1.**
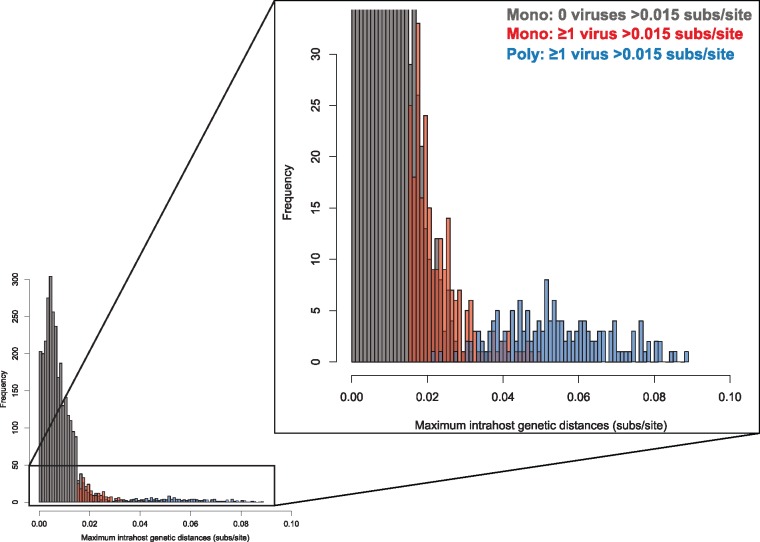
Maximum intra-host genetic distance. Color denotes group: gray are the 2,914 individuals with monophyletic virus in which no consecutive virus is >0.015 substitutions/site divergent; red are the 240 individuals with monophyletic virus in which ≥1 consecutive virus is >0.015 substitutions/site; blue are the 149 individuals with monophyletic virus in which ≥1 consecutive virus is >0.015 substitutions/site divergent.

Those 240 individuals who had highly divergent consecutively sampled viruses are similar to the extreme of the other 2,988 individuals with monophyletic virus (gray and red bars in Fig. 1). In contrast, virus from the 149 individuals with polyphyletic virus and probable superinfection formed a separate, more extreme distribution (blue bars in Fig. 1). The maximum genetic distance among these polyphyletic cases resembled random, within-Subtype B genetic distances in the US (Wertheim et al. 2017a). Individuals from all three groups had instances of within-host genetic divergence >0.03 substitutions/site, approaching random within subtype-B divergence. A similar pattern distinguishing these three groups can be seen in the mean within-host genetic distance (Supplementary Fig. S1).

### 3.4 Distinguishing individuals with monophyletic and polyphyletic viruses

The phylogenetic and genetic distance approach to characterizing superinfection is limited by the inherent difficulty in distinguishing within-host diversity from superinfection from another person with a closely related virus (i.e. superinfection from within a transmission cluster). Therefore, it is possible that the tail of the distribution of uppermost genetic distances for individuals with monophyletic virus is actually superinfection from a closely related source.

**Figure 2.**
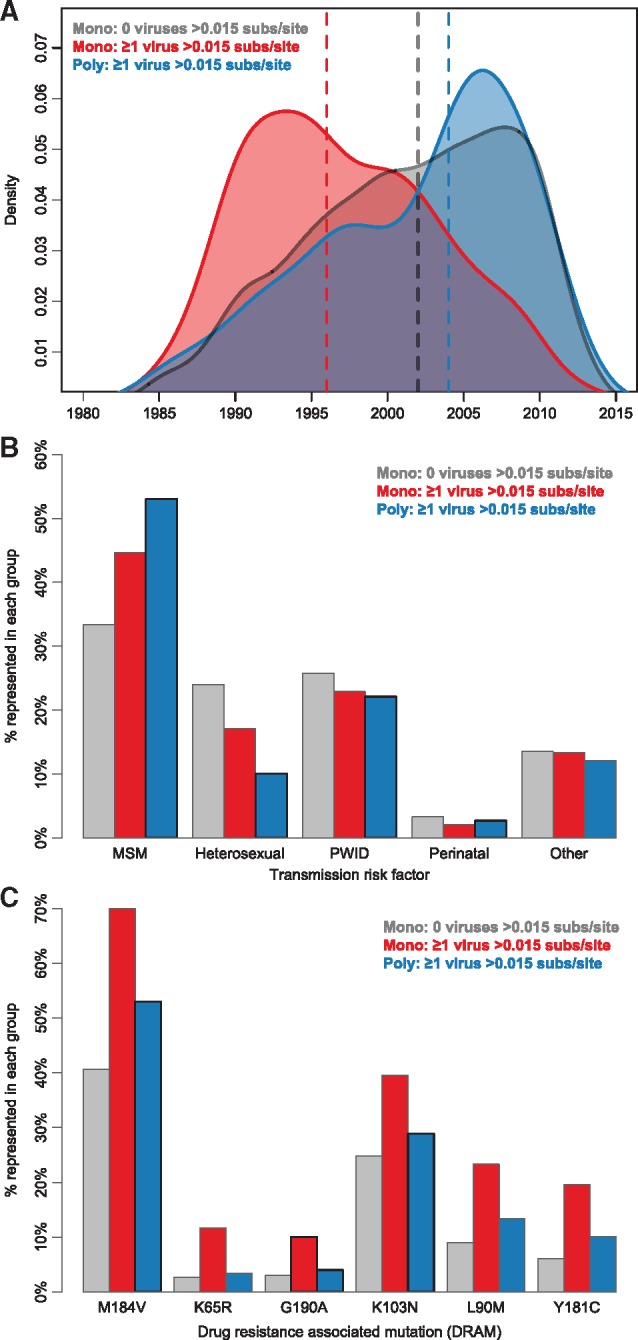
Comparison of individuals with monophyletic (mono) and polyphyletic (poly) viruses. (A) Density plots of year of diagnosis. Mean diagnosis year is shown with dashed lines. (B) Percentage composition of each group by transmission risk factor. (C) Frequency of different DRAMs in each group. Color denotes group: gray are the 2, 914 individuals with monophyletic virus in which no consecutive virus is >0.015 substitutions/site divergent; red are the 240 individuals with monophyletic virus in which ≥1 consecutive virus is >0.015 substitutions/site divergent; blue are the 149 individuals with monophyletic virus in which ≥1 consecutive virus is >0.015 substitutions/site divergent.

Individuals with monophyletic, but extremely divergent virus were typically diagnosed significantly earlier in time (earliest 25%: 1992; median: 1996; latest 75%: 2002) than individuals with either polyphyletic virus (earliest 25%: 1997; median: 2004; latest 75%: 2007) or monophyletic virus with no extremely divergent strains (earliest 25%: 1996; median: 2002; latest 75%: 2007) (*P* < 0.001; Table 1; Fig. 2A). Furthermore, individuals with polyphyletic viruses were significantly more likely to have identified as MSM (adjusted odds ratio [AOR] 2.16; *P* = 0.024) or PWID (AOR 2.23; *P* = 0.036) than individuals with extremely divergent consecutive viruses that were monophyletic (Table 1; Fig. 2B). These monophyletic individuals were more likely to have reported high-risk heterosexual activity or other risk factors. The proportion of PWID was not substantially different across these groups, which suggested that the significant AOR (Table 1) was attributable to early diagnosis years among PWID than non-PWID (median 1998 vs. 2003; Mann–Whitney U test; *P* < 0.001). DRAMs were significantly more common in individuals with monophyletic virus with extremely divergent, consecutively sampled virus than in individuals with monophyletic virus without extremely divergent virus (Table 1; Fig. 2C).

**Table 1.** Multinomial regression analysis of individuals who are either monophyletic with no consecutive virus >0.015 substitutions/site or polyphyletic with ≥1 consecutive virus >0.015 substitutions/site, compared with the reference group of individuals who are monophyletic with ≥1 consecutive virus >0.015 substitutions/site.

Attribute	Monophyletic with no consecutive virus >0.015 substitutions/site	Polyphyletic with ≥1 consecutive virus >0.015 substitutions/site
	AOR (95% CI)	AOR (95% CI)
Transmission risk factor		
MSM	0.58 (0.39–0.86)**	2.16 (1.11–4.22)*
Heterosexual	Ref.	Ref.
PWID	1.02 (0.66–1.59)	2.23 (1.05–4.72)*
Perinatal	2.31 (0.86–6.18)	4.33 (1.00–18.83)
Other	0.57 (0.34–0.94)*	1.20 (0.52–2.79)
Diagnosis year	1.09 (1.07–1.11)***	1.10 (1.07–1.14)***
DRAM		
M184V	0.45 (0.33–0.62)***	0.71 (0.45–1.11)
K103N	0.60 (0.45–0.80)**	0.68 (0.43–1.08)
Y181C	0.51 (0.34–0.77)**	0.79 (0.40–1.56)
K65R	0.38 (0.23–0.61)***	0.33 (0.12–0.91)*
G190A	0.62 (0.36–1.07)	0.67 (0.25–1.78)
L90M	0.50 (0.35–0.71)***	0.71 (0.40–1.28)

AOR, Adjusted odds ratio from multinomial regression analysis; CI, Confidence interval; MSM, Men who have sex with men; PWID, Persons who inject drugs; DRAM, Drug resistance associated mutation.

*** *P* < 0.001; ** *P* < 0.01; *P < 0.05.

### 3.5 Investigating the patterns of extreme within-host genetic divergence

To better understand the evolutionary patterns that gave rise to extremely divergent intra-host viral variants, we performed Bayesian molecular clock analysis on individuals who had monophyletic virus and a maximum genetic distance of at least 0.025 substitutions/site (the upper 2.5% tail of maximum intra-host divergence in people with monophyletic virus). We restricted this analysis to the eleven individuals with at least ten viral genotype sequences to more clearly understand patterns of viral genetic variation. Genotype sampling in these eleven individuals was dense over the observation period. The 3,303 individuals previously analyzed had a mean of 1.3 genotypes reported per person-year. Within these eleven individuals (denoted here as Cases A through K), there was an average of 2.2 viral genotypes per person-year (240 genotypes over 60.6 person-years; Table 2).

**Table 2.** Phylogenetic and genetic distance for eleven individuals with a maximum intra-host genetic distance >0.025 substitutions/site and ≥10 monophyletic viral genotypes.

Case	Diagnosis year	TMRCA		Maximum genetic distance (subs/site)	Years between first and last genotype	Years between diagnosis and last genotype	Viruses >0.015 subs/site from previous
		Median	95% HPD				
A	2002	1999	1994 − 2003	0.0292	4.4	8.9	0
B	1990	2004	2000 − 2007	0.0287	2.3	24.9	4
C	1984	1990	1984 − 1994	0.0375	7.9	23.8	5
D	2001	1998	1992 − 2002	0.0301	4.4	12.6	3
E	1998	2002	1998 − 2005	0.0399	5.4	17.2	1
F	1990	1993	1988 − 1996	0.0365	13.4	23.0	3
G	1996	2002	1998 − 2005	0.0270	4.1	16.5	2
H	1994	1992	1988 − 1996	0.0323	7.9	14.8	2
I	1995	1998	1994 − 2001	0.0316	3.7	16.6	2
J	2000	2004	2001 − 2007	0.0258	5.5	14.5	0
K	1990	1995	1990 − 1999	0.0400	1.6	22.1	3

TMRCA, Time of most recent common ancestor.

HPD, Highest posterior density.

The molecular clock analysis suggested that the extreme genetic distance observed in these eleven cases was consistent with their long duration of infection. In eight of the eleven cases, the 95% highest probability density for the inferred time of most recent common ancestor (TMRCA) included the year of diagnosis (Table 2). In three cases (Cases B, G, and J) the TMRCA was more recent than the year of diagnosis. In none of these cases did the TMRCA predate the year of diagnosis. However, we caution that date of diagnosis is necessarily the upper limit on the date of infection, which can preced the date of diagnosis by years. Further, the TMRCA should not *a priori* be expected to extend back to the date of infection.

**Figure 3.**
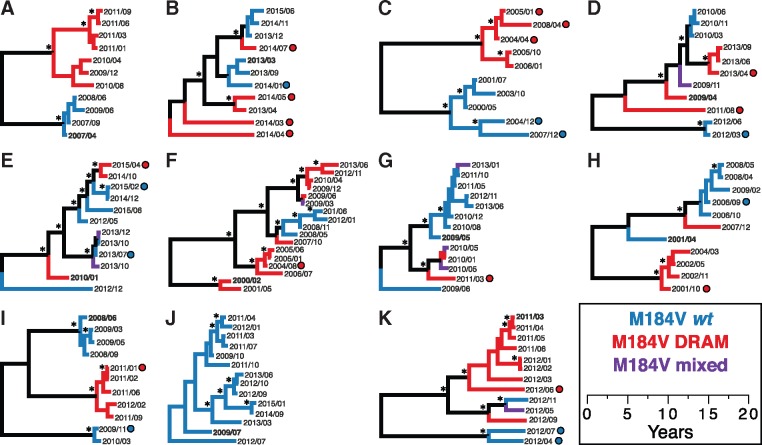
Maximum clade credibility trees from BEAST analysis. These eleven cases (A–K) were monophyletic in the phylogeny, have maximum genetic distance >0.025 substitutions/site, and at least ten reported viral genotypes. The earliest sampled (i.e. baseline) genotype is highlighted in bold. The height of the tips in these trees corresponds with date of sampling, and tips are labeled with year and month of sampling. Circles denote virus that is >0.015 substitutions/site from previously sampled genotype. All trees are shown on same time-scale. Branch color denotes drug resistance profile at M184V; clades are colored only when entire clade shares same profile. Asterisks indicate posterior support ≥0.90.

**Figure 4.**
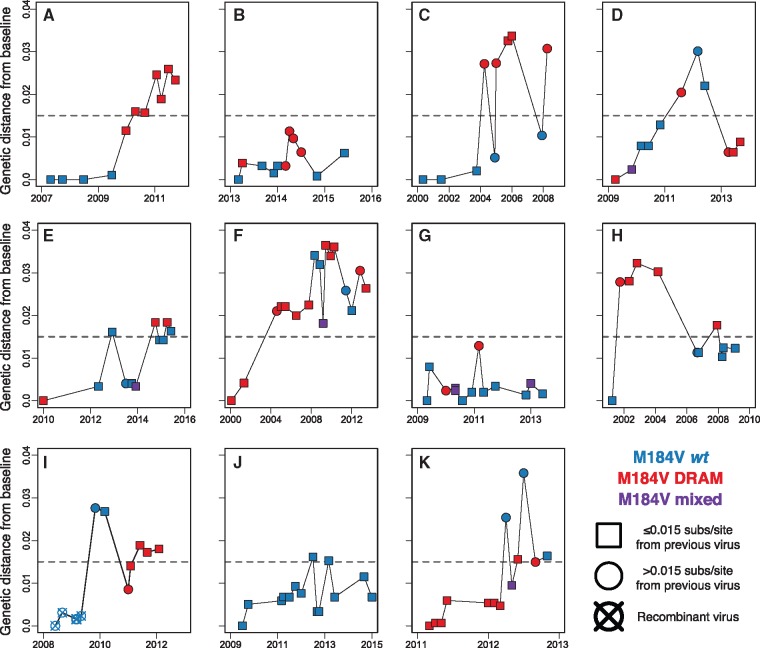
Longitudinal genetic distance from the earliest sampled (i.e. baseline) sequence. These eleven cases (A–K) were monophyletic in the phylogeny, have maximum genetic distance >0.025 substitutions/site, and at least ten reported viral genotypes. Node shape denotes the genetic relationship to the previous sequence. Note that the resolution of ambiguous bases can result in violations of the triangle inequality for genetic distance among viruses. Color denotes drug resistance profile at M184V. Dashed horizontal lines indicate 0.015 substitutions/site from baseline sequence.

Nine of these analyzed cases had a total of twenty-five instances of consecutively sampled viruses with >0.015 substitutions/site from the previous sequence (ranging between one and five instances per person) (Table 2; Figs 3 and 4). We note that in many instances, these highly divergent viruses alternate between resistant and wild-type mutations at M184V. Case C exhibits five such events, alternating between M184V-resistant and distantly related wild-type clades. We found evidence for recombination in the *pol* region in only one of these individuals (Case I; Fig. 3). Nonetheless, we also observed fluctuation between M184V resistant and wild-type virus in this same Case. Only Cases A and J did not have any consecutively sampled virus that was >0.015 substitutions/site divergent. Nonetheless, the total genetic divergence detected in each of these two cases was over 0.025 substitutions/site.

## 4. Discussion

We report the results of an investigation into longitudinal genetic variation in HIV *pol* genotypes within the US NHSS. We found 149 (4.2%) individuals infected with highly divergent (i.e. >0.015 substitutions/site), consecutively sampled viral genotypes that were polyphyletic in a large HIV phylogeny. Surprisingly, we found >1.6-times as many individuals (240; 7.3%) with highly divergent, consecutively genotyped viruses that were monophyletic in the HIV phylogeny. This latter group was distinct from individuals with probable superinfection, comprising people who were diagnosed, on average, nearly a decade earlier than inferred cases of superinfection. Furthermore, the maximum genetic distance within these individuals with extremely divergent, monophyletic virus closely resembled the maximum genetic distance observed in individuals without evidence of superinfection or highly divergent, consecutively sampled viral genotypes.

A phylogenetic examination of eleven cases exhibiting extremely divergent, monophyletic virus suggests that decade worth of viral diversity is maintained within individuals. Many of these individuals had been infected for over 20 years, and this level of divergence is consistent with evolutionary rate in this region of HIV-1 *pol*, of about 1 × 10^−^^3^ substitutions/site/year. However, this substitution rate is consistent with among-host evolutionary rates, and the intra-host substitution may be substantially faster (Lythgoe and Fraser 2012; Alizon and Fraser 2013; Landais et al. 2017). However, the unusually long duration of infection and the slowing of evolution due to ART (Kearney et al. 2014; Lorenzo-Redondo et al. 2016) make it difficult to determine the appropriate rate prior for these cases. Moreover, the long-duration over which these individuals were surveilled raises the potential for a downward bias in viral substitution rate, inflating the TMRCA estimates (Ho et al. 2011). Regardless of the exact rate of evolution, the breadth of this accumulated genetic diversity in the eleven cases investigated in depth here was often detectable in genotypes sampled over a span of only a couple years (see Cases B and K in Table 2).

The maintenance of such highly genetically divergent strains, though common in chronic infection of another RNA virus, Hepatitis C virus, (Gray et al. 2011, 2012; Raghwani et al. 2016), has not been previously described for HIV-1. Longitudinal studies of HIV genetic variation have focused on the *env* due to its rapid evolutionary rate and immunological importance (Shankarappa et al. 1999; Laird Smith et al. 2016; Landais et al. 2017). A comprehensive investigation into longitudinal viral diversity across the entire HIV genome by Zanini et al. (2015) found that the *env* region underwent more frequent selective sweeps than the rest of the genome, resulting in the frequent purging of genetic diversity in the *env* region. However, Zanini et al. also documented rapid increases and decreases in *pol* diversity, though not to the extent reported here. Moreover, the only individual in the Zanini study that had *pol* divergence from baseline that approached the levels reported here (>0.02 substitutions/site) was assumed to be an instance of superinfection. Importantly, the mono-infected individuals in the Zanini (2015) study were followed <10 years since diagnosis, less than half-as-long as most of the cases with extremely divergent viruses described here.

We must consider the possibility that, rather than these cases representing the maintenance of extremely divergent populations, they are actually the result of superinfection from within a close-knit transmission cluster. This possibility would indicate that superinfection from within a transmission cluster occurs far more frequently than superinfection from an unrelated strain. Less plausibly, this possibility suggests that superinfection from individuals with closely related strains occurs preferentially in individuals with significantly older diagnosis dates and preferentially occurs among people with heterosexual risk factors. Although it is likely that some fraction of the individuals with extremely divergent, monophyletic strains are actually the result of superinfection, the substantial differences in time since diagnosis the between these monophyletic and polyphyletic groups suggests a different mechanism behind their genetic variation profiles.

We note that in many of instances of oscillation between genetically diverged clades within monophyletically infected individuals in this study, these clades can be distinguished by the presence or absence of drug resistance at M184V in reverse transcriptase (Fig. 3). Resurgence of drug-resistant HIV from latently infected cells after treatment modification or drug recycling is a well-documented phenomenon (Kijak et al. 2002; Deeks et al. 2003; Joos et al. 2008; Little et al. 2008; Hedskog et al. 2010; Rocheleau et al. 2017). Different cellular reservoirs (e.g. peripheral blood mononuclear cells) often harbor distinct viral populations that could be the source of these re-emergent strains (Rozera et al. 2012). Rather than generating *de novo* mutations after the re-introduction of ART, pre-existing viral variants encoding drug resistance emerge into dominance. These pre-existing variants may also possess the necessary compensatory mutations to offset the fitness deficit arising from drug resistance mutations (Nijhuis et al. 1999; Gonzalez-Ortega et al. 2011). During this study, however, we did not have access to ART histories for any of these cases to determine if this resurgence correlated with changes or adherence to ART. Additionally, the M184 codon has biological importance beyond its potential for conferring drug resistance. This codon resides within a highly conserved sequence motif and is a known cytotoxic T-lymphocyte (CTL) epitope (Harrer et al. 1996). Therefore, genetic variants at this site are potentially subjected to dynamic CTL immune pressures as well as selection for and against drug resistance.

Conducting this study in a surveillance setting presents several limitations. Attribution of samples to a different individual or unintentional merging of two individuals within a surveillance database could artificially increase our estimates of superinfection. Moreover, poor quality sequencing of a single viral genotype could artificially increase intra-host diversity yet preserve monophyly. Furthermore, this investigation was limited to the analysis of bulk Sanger consensus sequences, which are routinely reported as part of HIV molecular surveillance in the US. The lack of population-level resolution inherent in consensus sequences prevents us from obtaining a clear picture of longitudinal intra-host diversity in these cases of interest. If similar cases of extremely divergent monophyletic viruses can be found in well-documented research cohorts, more in-depth investigations into the intra-host viral population dynamics will likely be possible.

Public health agencies within and outside the US are increasingly incorporating molecular sequence analysis into their HIV surveillance activities to identify growing transmission clusters (Poon et al. 2016; Monterosso et al. 2017). The discovery of individuals with extremely divergent viruses may complicate efforts to identify potential transmission links using Sanger sequencing. Genetic distance approaches for constructing molecular transmission clusters implicitly assume relatively low levels of intra-host HIV diversity (<1.5% nucleotide identity). Whether using the earliest sampled genetic (Oster et al. 2015; Wertheim et al. 2017b; Kosakovsky Pond et al. 2018; Wertheim et al. 2018) sequence or all available sequences for a given person (Poon et al. 2015, 2016), HIV molecular epidemiological methods need to account for the presence of individuals within clusters whose intra-host genetic variation is as great as random intra-subtype variation. Although this phenomenon appears relatively rare—given the number of people with decades-old diagnoses in the molecular surveillance database—a small number of problematic sequences can have large effects in genetic distance-based molecular transmission networks (Aldous et al. 2012; Kosakovsky Pond et al. 2018). Examination of viral populations within an individual at each specimen collection time point using next-generation sequencing may help to reveal these hidden variants and further our understanding of viral transmission dynamics.

## Supplementary Material

Supplementary Data

## References

[vey030-B1] Abecasis A. B., Vandamme A. M., Lemey P. (2009) ‘Quantifying Differences in the Tempo of Human Immunodeficiency Virus Type 1 Subtype Evolution’, Journal of Virology, 83: 12917–24. 19793809 10.1128/JVI.01022-09 PMC2786833

[vey030-B2] Aldous J. L. et al (2012) ‘Characterizing HIV Transmission Networks across the United States’, Clinical Infectious Diseases: An Official Publication of the Infectious Diseases Society of America, 55: 1135–43. 22784872 10.1093/cid/cis612 PMC3529609

[vey030-B3] Alizon S., Fraser C. (2013) ‘Within-Host and between-Host Evolutionary Rates across the HIV-1 Genome’, Retrovirology, 10: 49. 23639104 10.1186/1742-4690-10-49 PMC3685529

[vey030-B4] Deeks S. G. et al (2003) ‘Persistence of Drug-Resistant HIV-1 after a Structured Treatment Interruption and Its Impact on Treatment Response’, AIDS (London, England), 17: 361–70. 10.1097/00002030-200302140-00010 12556690

[vey030-B5] Drummond A. J. et al (2012) ‘Bayesian Phylogenetics with BEAUti and the BEAST 1.7’, Molecular Biology and Evolution, 29: 1969–73. 22367748 10.1093/molbev/mss075 PMC3408070

[vey030-B6] Gonzalez-Ortega E. et al (2011) ‘Compensatory Mutations Rescue the Virus Replicative Capacity of VIRIP-Resistant HIV-1’, Antiviral Research, 92: 479–83. 22027647 10.1016/j.antiviral.2011.10.010

[vey030-B7] Gray R. R. et al (2011) ‘The Mode and Tempo of Hepatitis C Virus Evolution within and among Hosts’, BMC Evolutionary Biology, 11: 131. 21595904 10.1186/1471-2148-11-131 PMC3112090

[vey030-B8] Gray R. R. et al (2012) ‘A New Evolutionary Model for Hepatitis C Virus Chronic Infection’, PLoS Pathogens, 8: e1002656. 22570609 10.1371/journal.ppat.1002656 PMC3342959

[vey030-B9] Harrer E. et al (1996) ‘Recognition of the Highly Conserved YMDD Region in the Human Immunodeficiency Virus Type 1 Reverse Transcriptase by HLA-A2-Restricted Cytotoxic T Lymphocytes from an Asymptomatic Long-Term Nonprogressor’, The Journal of Infectious Diseases, 173: 476–9. 8568316 10.1093/infdis/173.2.476

[vey030-B10] Hedskog C. et al (2010) ‘Dynamics of HIV-1 Quasispecies during Antiviral Treatment Dissected Using Ultra-Deep Pyrosequencing’, PLoS One, 5: e11345. 20628644 10.1371/journal.pone.0011345 PMC2898805

[vey030-B11] Hightower G. K. et al (2013) ‘HIV-1 Clade B Pol Evolution following Primary Infection’, PLoS One, 8: e68188. 23840830 10.1371/journal.pone.0068188 PMC3695957

[vey030-B12] Ho S. Y. et al (2011) ‘Time-Dependent Rates of Molecular Evolution’, Molecular Ecology, 20: 3087–101. 21740474 10.1111/j.1365-294X.2011.05178.x

[vey030-B13] Hu D. J. et al (2005). ‘Frequency of HIV-1 Dual Subtype Infections, Including Intersubtype Superinfections, Among Injection Drug Users in Bangkok, Thailand. AIDS (London, England), 19: 303-308. 15718841

[vey030-B14] Huerta-Cepas J., Serra F., Bork P. (2016) ‘ETE 3: Reconstruction, Analysis, and Visualization of Phylogenomic Data’, Molecular Biology and Evolution , 33: 1635–8. 26921390 10.1093/molbev/msw046 PMC4868116

[vey030-B15] Joos B. et al (2008) ‘HIV Rebounds from Latently Infected Cells, Rather than from Continuing Low-Level Replication’, Proceedings of the National Academy of Sciences of the United States of America, 105: 16725–30. 18936487 10.1073/pnas.0804192105 PMC2575487

[vey030-B16] Kearney M. F. et al (2014) ‘Lack of Detectable HIV-1 Molecular Evolution during Suppressive Antiretroviral Therapy’, PLoS Pathogens, 10: e1004010. 24651464 10.1371/journal.ppat.1004010 PMC3961343

[vey030-B17] Kijak G. H. et al (2002) ‘Origin of Human Immunodeficiency Virus Type 1 Quasispecies Emerging after Antiretroviral Treatment Interruption in Patients with Therapeutic Failure’, Journal of Virology, 76: 7000–9. 12072500 10.1128/JVI.76.14.7000-7009.2002 PMC136319

[vey030-B18] Koelsch K. K. et al (2003) ‘Clade B HIV-1 Superinfection with Wild-Type Virus after Primary Infection with Drug-Resistant Clade B Virus’, AIDS (London, England), 17: F11–6. 10.1097/00002030-200305020-00001 12700477

[vey030-B19] Koning F. A. et al (2013) ‘Dynamics of HIV Type 1 Recombination following Superinfection’, AIDS Research and Human Retroviruses, 29: 963–70. 23495713 10.1089/aid.2013.0009 PMC3653373

[vey030-B20] Kosakovsky Pond S. L. et al (2018) ‘HIV-TRACE (TRAnsmission Cluster Engine): A Tool for Large Scale Molecular Epidemiology of HIV-1 and Other Rapidly Evolving Pathogens’, Molecular Biology and Evolution, 35: 1812–9. 29401317 10.1093/molbev/msy016 PMC5995201

[vey030-B21] Laird Smith M. et al (2016) ‘Rapid Sequencing of Complete Env Genes from Primary HIV-1 Samples’, Virus Evolution, 2: vew018. 29492273 10.1093/ve/vew018 PMC5822884

[vey030-B22] Landais E. et al (2017) ‘HIV Envelope Glycoform Heterogeneity and Localized Diversity Govern the Initiation and Maturation of a V2 Apex Broadly Neutralizing Antibody Lineage’, Immunity, 47: 990–1003.e1009. 10.1016/j.immuni.2017.11.002 PMC5736302 29166592

[vey030-B23] Lemey P. et al (2005) ‘Molecular Footprint of Drug-Selective Pressure in a Human Immunodeficiency Virus Transmission Chain’, Journal of Virology, 79: 11981–9. 16140774 10.1128/JVI.79.18.11981-11989.2005 PMC1212611

[vey030-B24] Little S. J. et al (2008) ‘Persistence of Transmitted Drug Resistance among Subjects with Primary Human Immunodeficiency Virus Infection’, Journal of Virology, 82: 5510–8. 18353964 10.1128/JVI.02579-07 PMC2395184

[vey030-B25] Liu T. F., Shafer R. W. (2006) ‘Web Resources for HIV Type 1 Genotypic-Resistance Test Interpretation’, Clinical Infectious Diseases : an Official Publication of the Infectious Diseases Society of America, 42: 1608–18. 16652319 10.1086/503914 PMC2547473

[vey030-B26] Lorenzo-Redondo R. et al (2016) ‘Persistent HIV-1 Replication Maintains the Tissue Reservoir during Therapy’, Nature, 530: 51–6. 26814962 10.1038/nature16933 PMC4865637

[vey030-B27] Lythgoe K. A., Fraser C. (2012) ‘New Insights into the Evolutionary Rate of HIV-1 at the within-Host and Epidemiological Levels’, Proceedings of the Royal Society B: Biological Sciences, 279: 3367–75. 22593106 10.1098/rspb.2012.0595 PMC3385732

[vey030-B28] Martin D. P. et al (2010) ‘RDP3: A Flexible and Fast Computer Program for Analyzing Recombination’, Bioinformatics (Oxford, England), 26: 2462–3. 10.1093/bioinformatics/btq467 PMC2944210 20798170

[vey030-B29] Monterosso A. C. et al (2017) ‘Identifying and Investigating a Rapidly Growing HIV Transmission Cluster in Texas’, in Conference on Retroviruses and Opportunistic Infections (CROI). Seattle, WA.

[vey030-B30] Nijhuis M. et al (1999) ‘Increased Fitness of Drug Resistant HIV-1 Protease as a Result of Acquisition of Compensatory Mutations during Suboptimal Therapy’, AIDS (London, England), 13: 2349–59. 10.1097/00002030-199912030-00006 10597776

[vey030-B31] Oster A. M. et al (2015) ‘Using Molecular HIV Surveillance Data to Understand Transmission between Subpopulations in the United States’, Journal of Acquired Immune Deficiency Syndromes (1999), 70: 444–51. 26302431 10.1097/QAI.0000000000000809 PMC4878401

[vey030-B32] Poon A. F. et al (2016) ‘Near Real-Time Monitoring of HIV Transmission Hotspots from Routine HIV Genotyping: An Implementation Case Study’, The Lancet. HIV, 3: e231–8. 27126490 10.1016/S2352-3018(16)00046-1 PMC4853759

[vey030-B33] Poon A. F. et al (2015) ‘The Impact of Clinical, Demographic and Risk Factors on Rates of HIV Transmission: A Population-Based Phylogenetic Analysis in British Columbia, Canada’, The Journal of Infectious Diseases, 211: 926–35. 25312037 10.1093/infdis/jiu560 PMC4351365

[vey030-B34] Price M. N., Dehal P. S., Arkin A. P. (2010) ‘FastTree 2–Approximately Maximum-Likelihood Trees for Large Alignments’, PLoS One, 5: e9490. 20224823 10.1371/journal.pone.0009490 PMC2835736

[vey030-B35] Raghwani J. et al (2016) ‘Exceptional Heterogeneity in Viral Evolutionary Dynamics Characterises Chronic Hepatitis C Virus Infection’, PLoS Pathogens, 12: e1005894. 27631086 10.1371/journal.ppat.1005894 PMC5025083

[vey030-B36] Rambaut A. et al (2018) ‘Posterior Summarisation in Bayesian Phylogenetics Using Tracer 1.7’, Systems Biology, 67: 901–4. 10.1093/sysbio/syy032 PMC6101584 29718447

[vey030-B37] Ramos A. et al (2002) ‘Intersubtype Human Immunodeficiency Virus Type 1 Superinfection following Seroconversion to Primary Infection in Two Injection Drug Users’, Journal of Virology, 76: 7444–52. 12097556 10.1128/JVI.76.15.7444-7452.2002 PMC136380

[vey030-B38] Rocheleau G. et al (2017) ‘Longitudinal Trends of HIV Drug Resistance in a Large Canadian Cohort (1996-2016)’, Clinical Microbiology and Infection, 24: 184–91. 10.1016/j.cmi.2017.06.014 28652115

[vey030-B39] Ronen K. et al (2014) ‘HIV-1 Superinfection Is Associated with an Accelerated Viral Load Increase but Has a Limited Impact on Disease Progression’, AIDS (London, England), 28: 2281–6. 10.1097/QAD.0000000000000422 PMC4503239 25102090

[vey030-B40] Rozera G. et al (2012) ‘Ultra-Deep Sequencing Reveals Hidden HIV-1 Minority Lineages and Shifts of Viral Population between the Main Cellular Reservoirs of the Infection after Therapy Interruption’, Journal of Medical Virology, 84: 839–44. 22996031 10.1002/jmv.23292

[vey030-B41] Shankarappa R. et al (1999) ‘Consistent Viral Evolutionary Changes Associated with the Progression of Human Immunodeficiency Virus Type 1 Infection’, Journal of Virology, 73: 10489–502. 10559367 10.1128/jvi.73.12.10489-10502.1999 PMC113104

[vey030-B42] Smith D. M., Richman D. D., Little S. J. et al (2005) ‘HIV Superinfection’, The Journal of Infectious Diseases , 192: 438–44. 15995957 10.1086/431682

[vey030-B43] Smith D. M. et al (2004) ‘Incidence of HIV Superinfection following Primary Infection’, JAMA, 292: 1177–8. 15353529 10.1001/jama.292.10.1177

[vey030-B44] Smith D. M. et al (2005) ‘HIV Drug Resistance Acquired through Superinfection’, AIDS (London, England), 19: 1251–6. 10.1097/01.aids.0000180095.12276.ac 16052079

[vey030-B45] Stamatakis A. (2014) ‘RAxML Version 8: A Tool for Phylogenetic Analysis and Post-Analysis of Large Phylogenies’, Bioinformatics, 30: 1312–3. 24451623 10.1093/bioinformatics/btu033 PMC3998144

[vey030-B46] Struck D. et al (2014) ‘COMET: adaptive Context-Based Modeling for Ultrafast HIV-1 Subtype Identification’, Nucleic Acids Research, 42: e144. 25120265 10.1093/nar/gku739 PMC4191385

[vey030-B47] Tamura K., Nei M. (1993) ‘Estimation of the Number of Nucleotide Substitutions in the Control Region of Mitochondrial DNA in Humans and Chimpanzees’, Molecular Biology and Evolution, 10: 512–26. 8336541 10.1093/oxfordjournals.molbev.a040023

[vey030-B48] Wagner G. A. et al (2017) ‘Intrasubtype B HIV-1 Superinfection Correlates with Delayed Neutralizing Antibody Response’, Journal of Virology, 91: e00475–17 28615205 10.1128/JVI.00475-17 PMC5553187

[vey030-B49] Wagner G. A. et al (2014) ‘Incidence and Prevalence of Intrasubtype HIV-1 Dual Infection in at-Risk Men in the United States’, The Journal of Infectious Diseases, 209: 1032–8. 24273040 10.1093/infdis/jit633 PMC3952674

[vey030-B50] Wertheim J. O., Fourment M., Kosakovsky Pond S. L. (2012) ‘Inconsistencies in Estimating the Age of HIV-1 Subtypes Due to Heterotachy’, Molecular Biology and Evolution, 29: 451–6. 22045998 10.1093/molbev/msr266 PMC3258043

[vey030-B53] Wertheim J. O. et al (2016) ‘The International Dimension of the U.S. HIV Transmission Network and Onward Transmission of HIV Recently Imported into the United States’, AIDS Research of Human Retroviruses, 32: 1046–53. 10.1089/aid.2015.0272 PMC5067842 27105549

[vey030-B51] Wertheim J. O. et al (2017a) ‘Social and Genetic Networks of HIV-1 Transmission in New York City’, PLoS Pathogens, 13: e1006000. 28068413 10.1371/journal.ppat.1006000 PMC5221827

[vey030-B54] Wertheim J. O. et al (2017b) ‘Transmission Fitness of Drug-Resistant HIV Revealed in a Surveillance System Transmission Network’, Virus Evolution, 3: vex008. 28458918 10.1093/ve/vex008 PMC5399924

[vey030-B52] Wertheim J. O. et al (2018) ‘Growth of HIV-1 Molecular Transmission Clusters in New York City’, Journal of Infectious Disease, 218: 1943–53. 10.1093/infdis/jiy431 PMC6217720 30010850

[vey030-B55] Wheeler W. H. et al (2010) ‘Prevalence of Transmitted Drug Resistance Associated Mutations and HIV-1 Subtypes in New HIV-1 Diagnoses, U.S.-2006’, AIDS (London, England), 24: 1203–12. 10.1097/QAD.0b013e3283388742 20395786

[vey030-B56] Zanini F. et al (2015) ‘Population Genomics of Intrapatient HIV-1 Evolution’, Elife, 4: e11282. 26652000 10.7554/eLife.11282 PMC4718817

