# Supplementary material for: Maintenance and reappearance of extremely divergent intra-host HIV-1 variants
Source: Virus Evol. 2018 Dec 4;4(2):vey030. doi: 10.1093/ve/vey030 (PMC6279948; doi:10.1093/ve/vey030)
Supplement: Supplementary Data [file vey030_supp.zip › Wertheim_FigureS1.docx]

**
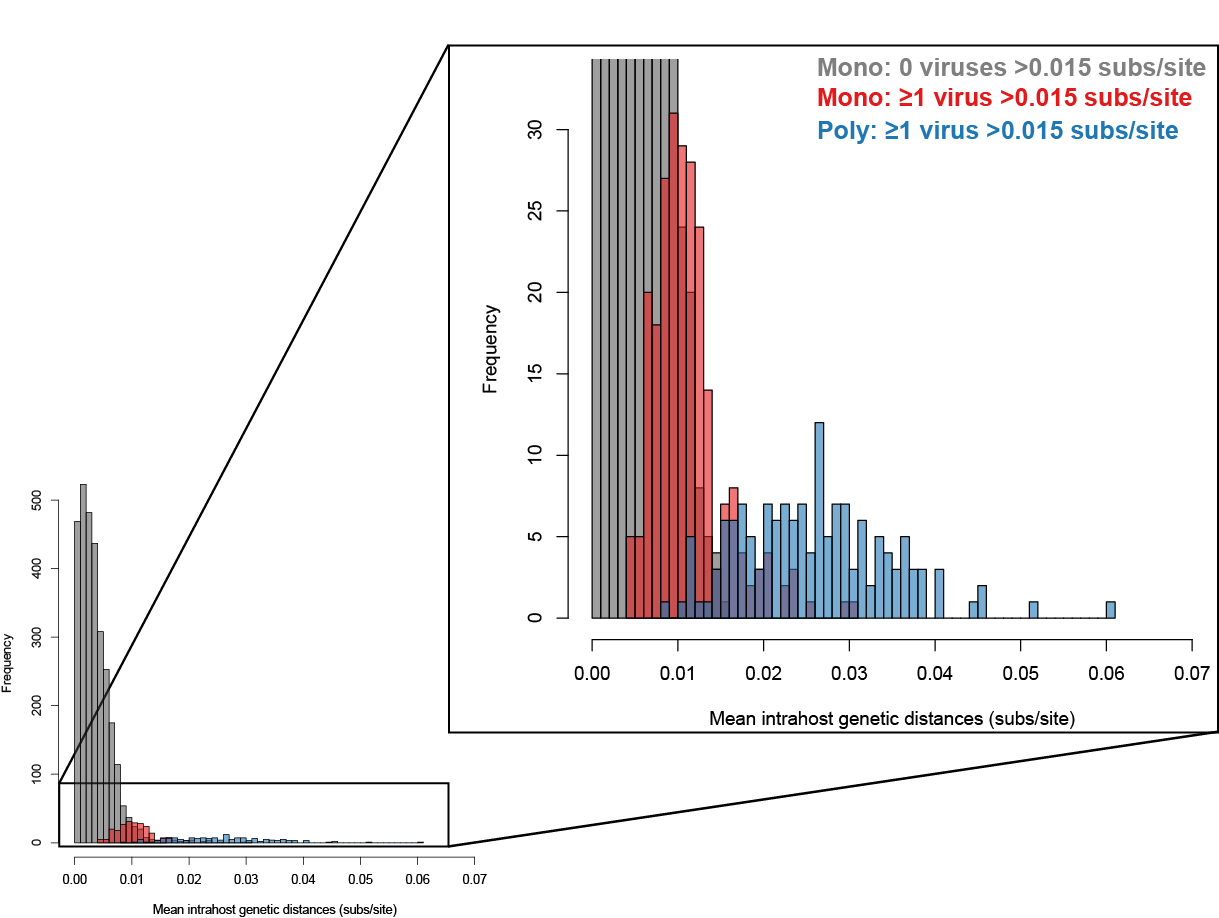
**

**Supplementary Figure 1. Mean intra-host genetic distance.** Color denotes group: gray are the 2,914 individuals with monophyletic virus in which no consecutive virus is >0.015 substitutions/site divergent; red are the 240 individuals with monophyletic virus in which ≥1 consecutive virus is >0.015 substitutions/site divergent from the previous genotype; blue are the 149 individuals with monophyletic virus in which ≥1 consecutive virus is >0.015 substitutions/site divergent.
