# Supplementary material for: Maintenance and reappearance of extremely divergent intra-host HIV-1 variants
Source: Virus Evol. 2018 Dec 4;4(2):vey030. doi: 10.1093/ve/vey030 (PMC6279948; doi:10.1093/ve/vey030)
Supplement: Supplementary Data [file vey030_supp.zip › ResponseToReviewers_20180817.docx]

Dear Dr. Pybus,

We thank you and Dr. Bedford for your helpful criticism and patience with this manuscript. We have modified the manuscript in response to your additional comments. Please find our responses below (**in bold**).

Cheers,

Joel Wertheim

Comments from the Editor-in-Chief:

1) In the Abstract and Intro, you state you investigate "the prevalence of superinfection". I'm not sure you measure that value directly. The first sentence of the Discussion is perhaps a better description of your study ("...an investigation into longitudinal genetic variation in HIV...."

**We agree with the Editor and have changed the sentence in the Abstract to read “We investigated the longitudinal intra-host genetic variation of HIV in over 3,000 individuals in the United States (U.S.) National HIV Surveillance System with at least four reported HIV-1 polymerase (*pol*) sequences.” The sentence in the introduction now reads “We investigated the longitudinal intra-host genetic variation of HIV, with the intent of characterizing cases of superinfection in a United States (U.S.) National HIV Surveillance System.” We feel it important to state our intent to characterize superinfection, as this intent informed the study design.**

2) Page 6, line 20: rephrase (e.g. "we imposed a highly informative prior distribution on the evolutionary rate parameter of the strict clock model, with a mean X and a low stddev of Y"

**We thank the Editor for their suggestion. The sentence now reads “…we imposed a highly informative prior distribution on the substitution rate parameter of the strict molecular clock model, with a mean of 1.22x10^-3^ substitutions/site/year and standard deviation of 1x10^-6^.”**

3) Page 9, line 35: yes, differences in rates are often phrased in terms of intra vs inter host. But there is also the "time dependence" effect, which means that rates are lower when rates are estimated from seqs with a wider range of sampling times. The two factors can be confounded, because intra host data is often sampled over shorter timescales. In your case, intra host data is sampled over a long timescale, so perhaps the time-dependence effects is in play. It might be worth considering and discussing this.

**We agree with the Editor on this point and thank him for reminding of us its importance. We now include a sentence in the discussion highlighting this issue: “Moreover, the long-duration over which these individuals were surveilled raises the potential for a downward bias in viral substitution rate, inflating the TMRCA estimates.”**

4) I now understand better that you are proposing co-circulation in hosts of highly divergent lineages, and that the single (Sanger consensus) sequence at each time point can switch from representing one lineage to another. In this context, it's perhaps relevant that you are looking at pol sequences. If I remember correctly, Richard Neher / Jan Albert et al.'s deep-sequencing of untreated within host HIV diversity revealed more "ladder like" trees for env, and more "structured" trees with multiple lineages for other HIV genes. I think you should interpret your results in the context of that study. If you collapse Richard/Jan's pol data to a single consensus sequence, do you get genetic distances similar to those observed in your dataset?

**We agree with the Editor that the genomic region analyzed may be relevance here. We have gone through the manuscript to remind the reader that we analyzed the pol region. Moreover, we have added an entire paragraph to the discussion section regarding this issue: “The maintenance of such highly genetically divergent strains, though common in chronic infection of another RNA virus, hepatitis C virus, (Gray, et al. 2011; Gray, et al. 2012; Raghwani, et al. 2016), has not been previously described for HIV-1. Longitudinal studies of HIV genetic variation have focused on the env due to its rapid evolutionary rate and immunological importance (Shankarappa, et al. 1999; Laird Smith, et al. 2016; Landais, et al. 2017). A comprehensive investigation into longitudinal viral diversity across the entire HIV genome by Zanini et al. (2005) found that the env region underwent more frequent selective sweeps than the rest of the genome, resulting in the frequent purging of genetic diversity in the env region. However, Zanini et al. also documented rapid increases and decreases in pol diversity, though not to the extent reported here. Moreover, the only individual in the Zanini study that had pol divergence from baseline that approached the levels reported here (>0.02 substitutions/site) was assumed to be an instance of superinfection. Importantly, the mono-infected individuals in the Zanini (2005) study were followed less than 10 years, less than half-as-long as most of the cases with extremely divergent viruses described here.”**

5) The explanation for not releasing data provided in the manuscript is a little cursory and the text in your reviewer responses is better. Please could you provide a fuller explanation in the main text.

**We agree and have incorporated our previous comments to the reviewers. The section now reads: “All data included in this paper were collected and analyzed as part of CDC routine surveillance activities. These data cannot be made publicly available; CDC is not permitted to share or distribute any surveillance data due to an assurance of confidentiality authorized under Section 308(d) of the Public Health Service Act (United States of America). Each state has primary authority for determining whether their laws and regulations permit the submission to GenBank or other open databases.”**
